# Structured pharmaceutical care improves the health-related quality of life of patients with asthma

**DOI:** 10.1186/s40545-017-0097-7

**Published:** 2017-02-07

**Authors:** Philip O. Anum, Berko P. Anto, Audrey G. Forson

**Affiliations:** 1 National Drug Information Centre, Ministry of Health, Accra, Ghana; 2 Department of Clinical and Social Pharmacy, Kwame Nkrumah University of Science and Technology, Accra, Ghana; 3 Department of Medicine, School of Medicine and Dentistry, University of Ghana, Accra, Ghana

**Keywords:** Asthma, Health-related Quality of Life, Pharmaceutical Care

## Abstract

**Background:**

Asthma as a chronic health condition can be controlled when in addition to clinical care, adequate education and support is provided to enhance self-management. Like many other chronic health conditions improved self-management positively impacts the health-related quality of life (HRQoL). It can therefore be said that a well-structured pharmaceutical care delivery that addresses the issues related to patient education and support towards self-management stands a good chance of positively impacting asthma control.

This study evaluated the impact of a structured pharmaceutical care delivery on asthma control.

**Methods:**

A prospective pre-/post- intervention study of a single cohort of 77 adult out-patients visiting specialist asthma clinics in Ghana were assessed for HRQoL and peak expiratory flow rates (PEFR) one month after pharmaceutical care intervention. Pharmaceutical care intervention covered education on the health condition, pharmacotherapy and self-management issues as well as correction of inhaler-use technique, where necessary and when to urgently seek medical care. The mean difference of the HRQoL and PEFR values were subjected to paired samples *t*-test analysis.

**Results:**

Delivery of a structured pharmaceutical care led to a significant improvement in asthma specific quality of life and PEFR. The mean paired difference of the HRQoL for a cohort of patients with asthma post- pharmaceutical care intervention was 0.697(95% CI: 0.490 - 0.900) at *t* = 6.85 (*p* < 0.05). The mean paired difference for PEFR post intervention was 17.533 (95% CI: 2.876 - 32.190) at *t* = 2.384 (p = 0.02).

**Conclusion:**

This study identified important challenges with both the pharmacologic and the non-pharmacologic management of adult asthma patients. Inadequate inhaler-use skills, widespread occurrence of preventable adverse events and irregular use of preventer medicines were prevalent among patients. At one month after pharmaceutical care intervention, patients with asthma in a cohort follow-up study showed significant improvements with regard to asthma-specific quality of life, peak flow rates and knowledge

**Trial registration:**

GHS-ERC: 08/9/11 of October 19, 2011.

## Background

In the year 2012, non-communicable diseases (NCDs) were responsible for 38 million (68%) of the 56 million global deaths as reported by the World Health Organization (WHO) [1]. This report classified more than 40% of these deaths as premature under the age of 70 years. In Ghana NCDs were reported to have accounted for 42% of total deaths of which about 2% were associated to chronic respiratory diseases [1].

Asthma is a chronic respiratory disease with a rising prevalence within the African region [2]. The Asthma Burden report has indicated that some 50 million cases of asthma are believed to prevail in Africa, with South Africa alone having a prevalence rate of about 8.1% [2]. The findings of the International Study of Asthma and Allergies in Childhood (ISAAC) report (2007) indicated that the international difference in asthma symptom prevalence has generally reduced, particularly in the 13–14 year age group and with decreases in prevalence in Western Europe [3]. The results however, indicated increases in prevalence in regions where prevalence was previously low, such as in Africa and parts of Asia [3]. These indications of growing trends of asthma in Africa were corroborated by Adeloye and colleagues [4] who also estimated a rising asthma prevalence in Africa,to be in the region of about 74 million to 119.3 million over the past two decades (1990–2010). The incidence rate of asthma in Ghana has however been estimated at 1.5/1000 per year by the WHO [5].

Notwithstanding these levels of occurrence, most health care systems are still organised around the acute care model which does not meet the management needs of patients with NCDs. The WHO-Innovative Care for Chronic Conditions report (2002) has suggested that supported self-management with regular follow-ups in addition to effective treatments improves the outcomes for chronically ill patients [6].

The main stay for asthma management that conforms to this innovative care for NCDs is the continuous monitoring of the patient for asthma control and therapy adjustments that are intended to minimize the symptoms regardless of the severity of the condition [7, 8]. Studies performed by Juniper and colleagues [9] have shown close correlation between the values of the Health-Related Quality of Life (HRQoL) of asthma patients and the levels of their asthma control. On the basis of these advances, various studies on asthma have used the HRQoL to assess the impact of interventions.

These trials, and especially those related to determination of effect of patient education by pharmacists on the outcomes of the disease have shown mixed results [10–16]. Some of these studies have indicated improvements in patient symptom scores [15], quality of life measurements, [15, 17–19] asthma severity [15, 17] and PFR [15, 17, 19]. The extent of the interventions was broad and may have introduced some bias into these studies. Various levels of bias could have been introduced from the drug therapy interventions and PEFR assessments and interpretations from the skills of the pharmacists at the multiple sites for the interventions. Some studies used only asthma severity [15] as primary outcomes measure, while others investigated only some aspects of asthma control [17]. Some of the studies protracted beyond 3 months, [15, 17–19] but did not reasonably resolve the issues of sustainability in such a chronic disease that is influenced by numerous intrinsic and extrinsic factors. The GINA [7] recommendations with regards to continuous monitoring and adjustments of therapy towards asthma control should apply here for pharmacy interventions as well.

Furthermore most of these studies used case–control methods in community settings where the participants in the “case” group cannot be totally isolated from those in the “control” group of the study. In cases where education impacts outcome, then it must be ensured that there is no exchange between the “case” and “control” participants.

It is for these reasons that this researchers opted for a single cohort study and fewer sites to allow only two trained pharmacists to undertake the interventions. The interventions were also only focused on patient understanding of the disease condition, the medications and the appropriate use of the prescribed medications. The study employed the use of the HRQoL instrument as the primary outcome measure of asthma control. Secondary outcomes were the measurements of PEFR and inhalation technique scores. This cohort trial was organised to study the hypothesis that provision of organised pharmaceutical care that focussed on patient education on prescribed medications, would improve asthma control in adult patients.

## Methods

### Design

A prospective pre-/post- intervention study of a single cohort of adult out-patients visiting specialist asthma clinics in Ghana were assessed for HRQoL and peak expiratory flow rates (PEFR) at baseline and one month after pharmaceutical care intervention. Pharmaceutical care intervention covered education on the health condition, pharmacotherapy and self-management issues as well as correction of inhaler-use technique, where necessary and self-referral for early unscheduled review for worsening asthma condition. The mean differences in HRQoL and PEFR values were subjected to a paired samples *t*-test analysis.

### Setting

Four out-patient clinical sites were used in the study. Two were teaching hospitals that managed referral cases from various health institutions and run specialist asthma clinics once a week. The two other hospitals were not teaching hospitals, but one runs specialist asthma clinics. All the clinical sites were located within the southern and middle belts of Ghana.

### Sample-size estimation

Assuming a mean HRQoL difference size of 0.5 (the minimum important difference of clinical significance) [20] and a standard deviation of HRQOL score of 0.8 from previous studies in the literature, the calculated standardized effect size (the mean difference / standard deviation) was estimated to be approximately 0.6. To estimate the sample size to test the hypothesis, a power (β) of 0.9 and the level (two-sided) of statistical significance (α) of 0.05 were set. Using these settings, the estimated sample size required for the cohort, when using the paired samples *t*-test to compare means of continuous variables to determine the minimum important difference of 0.5 was 60 patients. A convenience sample of 92 patients was used recruited for the study.

### Data collection instrument

Two sets of data collection instruments were used in this study. The Model Pharmaceutical Care Instrument was used to collect data on participant demographics, medication profile, inhaler-use technique, perceived adverse medication events, peak expiratory flow rates symptoms and environmental trigger factors. This was used to guide participant education. Review of this data identified care-needs of every participant in the study. Care needs addressed included: understanding of disease condition and the role of the various medications; inhaler-use technique; issues of medication adherence; availability of medications; management of trigger-factors; management of adverse events, and peak flow meter assessment of lung function.

The standard Asthma Quality of Life Questionnaire (AQLQ(S)) and the Peak Expiratory Flow Meter (“Airzone” flow meter by Clenment Clarke International) were used to assess participant HRQoL and lung function pre and post pharmaceutical care intervention..It was adopted to measure the functional problems (physical, emotional, social and occupational) that are most troublesome to adults (17–70 years) with asthma. The AQLQ(S) is an asthma-specific instrument that had been validated in clinical trials [21]. The AQLQ(S) contains 32 questions (items) comprising four domains: Activity Limitations, Asthma Symptoms, Emotional Function and Environmental Exposure. Each item was scored based on a 2-week recall of activities on a 7-point Likert scale with Point 1 indicating severe impairment and point 7 indicating no impairment. The overall score for HRQoL was the mean score of the 32 items.

A change in mean overall or domain score of 0.5 had been shown to represent the smallest change of importance to the patient (the “minimal important difference”), and a change in score of 1.0 represents a moderate change [20]. However, a maximum overall AQLQ(S) score of 7 represents no impairment in QOL due to asthma, and scores approaching 7 imply a minimal impact of asthma on QOL.

### Ethical clearance

Having obtained an ethical clearance from the Ghana Health Service (GHS-ECH: 08/9/11) and participant consent, ninety two (92) adults with symptomatic asthma, without other active co-morbidities, who visited the out-patient clinic, were recruited into the study.

### Data collection and intervention

Asthma patients attending regular clinical review in 4 selected hospitals located in the middle and southern belts of Ghana were recruited into the study. Eligibility for participation was limited to patients aged 17–70 years who have been medically diagnosed with asthma and had no active co-morbidities. Excluded in the study were patients whose medications were changed in their previous review visit prior to the recruitment. After giving their consent to participate in the study, a convenience sample of 92 patients was selected by selecting 10 patients out of a mean attendance of 30 patients per week from 4 hospitals for a period of 12 weeks. These participants were administered with the baseline questionnaires and provided educational intervention. These participants were followed up one month after the interventions. Fifteen (15) participants were lost to follow up. They did not show up for the post-intervention assessments. Therefore data for a total number of 77 patients from the baseline intervention were used for analysis.

Two clinical pharmacists, who were trained for the study, undertook the patient assessment and provided educational intervention. The HRQoL instrument was self-administered by participants. All other participant data was collected with the “Model Pharmaceutical Care” forms. The best of 3 PEFR assessments were entered for each participant. The total score for inhaler-use technique was collated for each participant. Inhaler-use technique score was the sum of one (1) point per each correct step undertaken by participant and observed by the study pharmacist. The maximum total of 7-points score marks were based on the extension of the manufacturers’ inhalation steps as found in product leaflets. Based on their inhaler-use technique scores, participants were coached to perform all the steps correctly. The “Model Pharmaceutical Care” forms were used to administer questionnaire on “perceived” environmental trigger factors on individual participant’s asthma and adverse effects from their prescribed medications. The impact of asthma with regards to airway narrowing and the roles of “reliever” and “preventer” medicines on the airways were explained to each participant. Participant’s knowledge, PEFR and HRQoL were consecutively re-assessed in one month after the provision of the interventions.

### Data analysis

Data from the study were subjected to both descriptive and inferential statistical analysis. Data were analyzed to generate various tables and charts to demonstrate similar themes, frequencies, trends and number counts under the following themes:

- Baseline Characteristics of Asthma Out-patient Clinic Participants
- Comparison of Asthmatic Patients’ Characteristics Post Pharmaceutical Care Intervention

Data collected demonstrated normal distribution with same values for central tendency. Data were therefore analyzed to test the study hypothesis using a paired samples *t*-test; the mean differences in the baseline and post pharmaceutical care intervention HRQoL were analyzed.

## Results

### General background characteristics at baseline

Study participants were predominantly female (61%) and had a mean age of 46 (±15) years. The mean peak expiratory flow rate was 284(±103) for the 77 participants. Most participants in the study had a minimum of high school education (97.5%) as in Table 1. The daily symptoms occurrence (11.7%) and the night-times awakenings (16.9%) frequencies were similar to reliever medication usage rates (18.2%), however there was a high irregular use (43.5%) of preventer medications (Table 1). Many participants (42.9%) reported one form or the other of various adverse events perceived to be associated to their asthma medicines.

**Table 1:** Background characteristics of participants at baseline

	Participants %
Education (*n* = 77)	
Primary School	2.6
High School	48.1
Tertiary	49.4
Symptoms Occurrence (*n* = 77)	
Less than or 2 days in a week	71.4
More than 2 days per week but not daily	16.9
Daily occurrence	11.7
Night-time Awakenings (*n* = 77)	
Less than two times in a month	67.5
Three to four times in a month	15.6
More than once in a week, but not every night	16.9
Use of Reliever Medications for Control (*n* = 77)	
Less than or 2 days in a week	58.4
More than two days in a week but not daily	23.4
Daily use	18.2
Adherence to Prescribed Preventer Medications (*n* = 69)	
Regular usage	56.5
Irregular usage	43.5
Adverse Drug Events Reported (*n* = 77)	
Yes	42.9
No	57.1
Reported Adverse Events with Preventer Medications (*n* = 69)	
Regular Preventer Medicine Usage with Adverse Events	48.7
Regular Preventer Medicine Usage with No Adverse Events	51.3
Irregular Preventer Medicine Usage with Adverse Events	40.0
Irregular Preventer Medicine Usage with No Adverse Events	60.0

### Incidence of adverse events

Participants reported of widespread incidence of adverse drug events perceived to by associated with their asthma medications. Coughs and dry mouth were the most frequently reported incidence of events (Table 2).

**Table 2:** Incidence of adverse events reported by participants

Adverse Events	Number of Participants	Adverse Events	Number of Participants
Coughs	8	Headache	2
Dry Mouth	6	Palpitations	1
Light Headedness	3	Sweating	1
Chest Burns	3	Phlegm	1
Sore Throat	3	Nasal Congestion	1
Itching	3	Blurred Vision	1
Sneeze	2	Pins & Needles	1
Nausea	2	Neck Pains	1

### Participants’ inhaler medication profile

Almost all participants (96%) used Salbutamol inhalers. In addition, Budesonide/formoterol (51%) and Fluticasone/salmeterol (31%) inhalers were used in various combinations (Table 3).

**Table 3:** Participant asthma medication profile

Inhaled Medication(s)	Number of Participants (%)	Patients on Prednisolone with Inhaler Medication(s)
Salbutamol Only	5 (6.5)	-
Salbutamol + beclomethasone	1 (1.3)	-
Salbutamol + pulmicort	8 (10.4)	1
Salbutamol + budesonide/formoterol	37 (48.1)	2
Salbutamol + fluticasone/salmeterol	22 (28.6)	3
Budesonide/Formoterol only	2 (2.6)	-
Fluticasone/salmeterol only	1 (1.3)	-
Salbutamol + Fluticasone/salmeterol + ipratropium	1 (1.3)	1

Table 4 presents the number of years participants had used various inhaler types. While 42% of participants had used “Reliever” inhalers for 10 years or more, only 4% of participants had used “Preventer” inhaler for that long. However, 55% of participants had used a “Preventer” for a minimum of one year. Some 6 participants on “Reliever” inhalers and 7 other participants on “Preventer” inhalers could not recollect for how long they had used these medicines.

**Table 4:** Participant inhaler medication usage

Years of Inhaler Usage	Participant’s Inhaler Type (*n* = 77)	
	Reliever (%)	Preventer (%)
≥10 years	31 (42.3)	3 (3.9)
6 –9 years	8 (10.4)	7 (9.1)
1-5 years	27 (35.1)	42 (54.5)
≤1 year	5 (6.5)	18 (23.4)
Cannot recollect	6 (7.8)	7 (9.1)

### Participants’ inhaler-use technique

Baseline inhaler-use technique score captured using an inhaler-use technique assessment questionnaire, shown in Table 5 indicate that only 12% and 17% of participants on dry powder inhalers (DPI) and metered dose inhalers (MDI) respectively scored the maximum score of 7 points. The mean inhaler-use technique scores were 4.66 (1.3) for the use of DPIs and 5.03(1.43) for the use MDIs.

**Table 5:** Participant inhaler-use technique score

	Inhaler-use Technique Score (7-point scale)								
Inhaler Type	1	2	3	4	5	6	7	*Percentage with Adequate Technique*	*Mean Score (SD)*
DPI^a^ (*n* = 66)	0	2	10	23	16	9	8	12.12	4.66(1.3)
MDI^b^ (*n* = 63)	0	4	3	13	19	13	12	17.46	5.03(1.43)

^a^ *PDI* dry powder inhaler, ^b^ *MDI* metered dose inhaler

### Environmental trigger-factors affecting participants

Dust (78%), perfume/and strong scents (78%) and smoke (70%) were the most commonly mentioned environmental trigger-factors that worsened the asthma of participants (Table 6). Other trigger-factors like room air-conditioning, fresh cut grass, and alcohol and beer intake affected about 5% of participants.

**Table 6:** Number of participants affected by environmental trigger-factors

Trigger-factor type	Participants Affected (*n* = 77)	Frequency (%)
Dust	60	77.92
Perfume /Scent	60	77.92
Smoke	54	70.13
Food / Spices	21	27.27
Weather Changes	19	24.68
Stress / Tiredness/Emotions	14	18.18
Cold Drinks	11	14.29
Others (beer, fresh cutting grass, alcohol & air-conditioner)	4	5.19

### Post-pharmaceutical care comparison of participant characteristics

The quantum of change in HRQoL is displayed in Table 7. About 56% of participants had a positive change in HRQoL, while 9% had a negative change after the pharmaceutical care intervention. However, about 35% of participants had changes that were not significant.

**Table 7:** Quantum changes in quality of life post-pharmaceutical care

Change	Health Related Quality of Life (*n* = 77)						
Quantum	−3	−2	−1	0	1	2	3
Range	−3.49-(−2.50)	−2.49- (−1.50)	−1.49- (−0.50)	−0.49-0.49	0.50-1.49	1.50-2.49	2.50-3.49
Number of Participants (%)	0 (0.00)	1 (1.30)	6 (7.79)	27 (35.06)	29 (37.66)	12 (15.58)	2 (2.60)
Change rate (%) in HRQoL	7 (9.09)			27(35.06)	43(55.84)		
	Negative Change			No Change	Positive Change		

From Table 8, the paired mean difference values for the HRQoL and all the domains were above 0.5, the mean important difference. The overall paired mean difference for the HRQoL at 95% CI was 0.697 (0.490-0.900) at 95% CI, with a T- value of 6.845 (2-tailed *p* < 0.001). The paired mean difference at 95% CI was highest for the Symptoms domain 1.134 (0.910 – 1.360) and lowest for the Activity Limitation domain 0.548 (0.340 - 0.760).

**Table 8:** Paired mean difference statistics of the health related quality of life of participants after intervention

Health-Related Quality of Life Domain		Mean (SD)	Paired Differences			T	Df	Sig. (2-tailed)
			Mean (SD)	*95% CI of the Difference*				
				Lower	Upper			
HRQoL	Pre-	4.15 (1.132)	0.697 (0.891)	0.490	0.900	6.845	76	0.000
	Post-	4.85 (1.271)						
Activity Limitation	Pre-	4.37 (1.143)	0.548 (0.921)	0.340	0.760	5.256	76	0.000
	Post-	4.92 (1.224)						
Symptoms	Pre-	3.998 (1.086)	1.134 (0.985)	0.911	1.358	10.104	76	0.000
	Post-	5.132 (1.304)						
Emotional Function	Pre-	3.907 (1.453)	0.714 (0.938)	0.501	0.927	6.684	76	0.000
	Post-	4.621 (1.583)						
Environmental Stimuli	Pre-	3.289 (1.562)	0.776 (1.099)	0.526	1.025	6.195	76	0.00
	Post-	4.065 (1.685)						

The mean (PEFR) at baseline was 266.5 L/min (SD 85.9), which increased to 284.0 L/min (SD 103.3) after the intervention (Table 9). The paired mean difference between baseline and post-pharmaceutical care intervention PEFR was 17.5 L/min. (95% CI: 2.876-32.190, *p* < 0.05).

**Table 9:** Paired mean difference statistics of peak expiratory flow rate after intervention

	Mean (SD) L/min.		Paired Differences			T	Df	Sig. (2-tailed)
			Mean (SD) L/min.	*95% CI of the Difference*				
				Lower	Upper			
Peak Flow Rate	Pre-	266.467 (85.857)	17.533 (63.705)	2.876	32.190	2.384	74	0.020
	Post-	284.000 (103.294)						

## Discussion

Overall participant Inhaler-use technique assessments revealed low scores. Over half of participants in this study had used Salbutamol metered-dose inhaler for more than 5 years, and yet very few had adequate inhaler-use technique to either the dry powder type of inhalers or the metered dose type of inhalers. It is possible that most of the participants had progressed up the therapeutic steps to the use of Salbutamol with Budesonide/formoterol or Fluticasone/salmeterol (step 3) as a result of poor asthma control due to inadequate use of inhalers. In conformity with third Expert Panel Review (EPR-3, 2007) and Global Initiative for Asthma (GINA, 2014) recommendations, usually after a 3-month period of well-controlled asthma there should have been a step down in therapy, [7, 21] but only 12% of patients were on ICS inhalers alone as controller medication. Poor inhaler-use technique and /or poor compliance could be the reason why patient’s asthma was not stable enough to be stepped down. The GINA guidelines have suggested that before increasing pharmacologic therapy, one must first consider poor inhaler technique, poor adherence and co-morbidities as targets for intervention [7].

The degree of poor inhaler-use technique identified among study participants indicates that not enough consideration has been given to the role of inhaler-use technique in patient management. It further suggests that there is insufficient patient education and counselling at the clinic and at the pharmacy, and that any education and counselling provided to the study participants in the past clearly did not adequately impact on their skills, or its impact might have diminished with time.

The common major environmental trigger-factor affecting participants in this study were exposure to dust, smoke and perfume or strong scents, which suggests that another major need for intervention is education on the improved control of environmental triggers by patients within their environment.

Previous studies by Mangiapane,and colleagues [18], using a single cohort design, observed significant changes in both HRQoL, and PEFR, among others. This study also, using a single cohort design found significant changes in the HRQoL and the PEFR. Whilst more than half of the study participants’ perceived improvements in HRQoL, 9% of them perceived worsened HRQoL and 35% did not perceived any changes in their HRQoL. Though no factual reasons could be assigned immediately, issues related to patient adherence to inhaler medications and the influence of environmental trigger-factors may have played a role. However, the deterioration and the non-significant change in HRQoL might also be explained by the “phenotype of asthma theory”. The EPR-3 [8] suggests that very specific patterns of inflammation exist in some individuals that require different treatment approaches in addition to the usual steroid-based anti-inflammatory treatment.

The paired mean difference values for the overall general HRQoL and its various domains were both clinically and statistically significant, showing benefit of the pharmaceutical care intervention to the patients in all aspects of their quality of life. The Symptoms domain had the highest mean change difference of 1.134 (95% CI: 0.910 - 1.360) and the Activity Limitation had the lowest of 0.548 (95% CI: 0.340 – 0.760). This is an indication that the various domains were impacted to different extents within the composite overall HRQoL. Barbanel and colleagues [13] did not use the HRQoL as the outcomes measure when they studied the impact of pharmacists’ intervention on asthma patients. They used asthma symptoms and observed significant improvements. This study also observed more significant improvements within the symptoms domain than the other various domains that constituted the composite HRQoL.

However, Mancuso and colleagues [22] found out that these favourable results from pharmacy interventions tended to wane with time if follow-ups were not pursued with participants, as may have been the case with our patients.

This study observed improvements in participants’ values at the end of the intervention as with their HRQoL.when PEFR measurements were used as a secondary measure of asthma control. However, analytical exploration of the values indicated only a weak positive correlation between the changes in the overall HRQoL and PEFR from baseline as a result of the intervention. Previous studies by [15, 19] among others, also observed significant improvements in the PEFR within the intervention groups.

PEFR are known to have an a diminished correlation with the FEV1 in patients with asthma, [23] however the EPR- 2007 recommends PEFR monitoring, using the patient’s best peak flow in an asthma action plan [21]. The weak correlation between the PEFR with the HRQoL therefore is a reflection of their controversial role in assessing airway obstruction rather than voluntary effort and muscular strength of the patient [23].

Outstanding are the economic implications of such intervention on the cost of asthma management. Further to any clinical significance of educational intervention, patients and health insurance schemes might make savings on asthma-related health care, cost of medications, hospitalization and emergency department visits as envisaged by [24–26]. Future studies may be needed to cost and reflect in monetary terms the savings from the impact of educational interventions in asthma.

### Limitations to the study

This study has some limitations. First, the duration of the study did not allow for the participants to be followed-up further to assess duration of the impact of the intervention or to ascertain whether the therapies of the participants with significant improvements in HRQoL were subsequently adjusted in the post- intervention months. The design of this study did not factor duration of impact of the intervention since asthma is a dynamic condition that would require continuous re-evaluation and further education in conformity to the recommendations of GINA and the EPR. Secondly, the AQLQ used in the study to assess HRQoL were not translated into any of the local languages for patients who may not be able to read and write English and thereby limited the scope of patient participation. Furthermore the study covered the southern and middle belts of the country and that may to some extent also limit the overall generalizability of the findings.

## Conclusion

This study identified important challenges with both the pharmacologic and the non-pharmacologic management of adult asthma patients. Inadequate inhaler-use skills, widespread occurrence of preventable adverse events and irregular use of preventer medicines were prevalent among patients. At one month after pharmaceutical care intervention, patients with asthma in a cohort follow-up study showed significant improvements with regard to asthma-specific quality of life, peak flow rates and knowledge.

### Significance of pharmaceutical services in the hospital

A well-structured pharmaceutical care delivery in the hospital would contribute to patient knowledge and management and eventually improve the health-related quality of life of asthma patients. This study has shown that hospital Pharmacists would need to be properly trained to assess patients with asthma and to provide the needed education to address their individual challenges associated with the condition and the management.

## Abbreviations

AQLQ(S): Standardized asthma quality of life questionnaire
DPI: Dry powder inhaler
EPR: Expert panel report
GHS: Ghana Health Service
GINA: Global initiative for asthma
HRQoL: Health related quality of life
MDI: Metered dose inhaler
NCD: Non-communicable disease
PEFR: Peak expiratory flow rate
WHO: World Health Organization
WHO-ICCC: World Health Organization-innovative care for chronic conditions
WHOQOL: World Health Organization Quality of Life
